# Pulmonary Langerhans Cell Histiocytosis Presenting as Multiple Obstructing Bronchial Lesions and Pulmonary Masses

**DOI:** 10.1111/crj.70194

**Published:** 2026-05-12

**Authors:** Jingyuan Fan, Chen Liu, Zhen Yang, Danxiong Sun

**Affiliations:** ^1^ Department of Respiratory and Critical Care Medicine The First People's Hospital of Yunnan Province Kunming China

**Keywords:** obstructing bronchial lesion, pulmonary Langerhans cell histiocytosis, pulmonary mass

## Abstract

Pulmonary Langerhans cell histiocytosis is a rare lung disease, which was previously classified as interstitial lung disease but is now classified as a myeloid neoplasm. The obstructing tracheobronchial lesion or mass may rarely be the manifestation of the disease. We describe a case of pulmonary Langerhans cell histiocytosis presenting as multiple obstructing bronchial lesions and pulmonary masses in a 36‐year‐old man. This case represents an extremely rare manifestation of pulmonary Langerhans cell histiocytosis.

## Graphical abstract

We describe a case of PLCH presenting as multiple obstructing bronchial lesions and pulmonary masses in a 36‐year‐old man. To our knowledge, this is the first report of PLCH presenting as bilateral hilar masses and airway obstructive lesions.

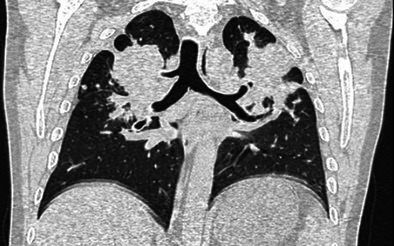

**Abbreviations**
CT: computed tomography
FDG: fluorodeoxyglucose
IFN: interferon
LCH: Langerhans cell histiocytosis
PLCH: pulmonary Langerhans cell histiocytosis
PET/CT: positron emission tomography/computed tomography
SUV: standardized uptake value

## 1 Introduction

Pulmonary Langerhans cell histiocytosis (PLCH) is a rare myeloid nonmalignant neoplasm with an inflammatory component. The most common CT findings were nodules and cysts, which are usually predominant in the upper and middle lung fields, with relative sparing of lung bases. Pulmonary mass and obstructing tracheobronchial lesion are the rare manifestations. We report a case of PLCH presenting as multiple obstructing bronchial lesions and bilateral hilar masses.

## 2 Case Report

A 36‐year‐old man was admitted to the hospital with repeated cough, blood‐stained sputum, and exertional dyspnea for about 18 months. About 18 months ago, the patient began to have productive cough with blood‐stained sputum and exertional dyspnea, which occurred after he climbed four flights of stairs. Computed tomography (CT) scan revealed multiple masses and consolidations in both lungs in a local hospital. Bronchoscopy was performed, and a small amount of bloody sputum was found at the right upper lobe bronchus without obstructing tracheobronchial lesions. He was hospitalized with a presumed diagnosis of pneumonia and treated empirically with moxifloxacin and methylprednisolone. After discharge, he had fever for about 10 days, and the highest body temperature was 39°C. Afterwards, the patient continued to cough repeatedly, with blood in the sputum and exertional dyspnea. He did not report weakness, anorexia, night sweats, weight loss, or chest pain. He was a social drinker and current smoker of approximately 18 cigarettes per day for 15 years, working as a freelancer, with no occupational dust or gas exposure and no significant medical history.

His admission vital signs were normal. He had no peripheral enlarged lymph nodes. On auscultation, the breath sounds were mildly diminished bilaterally. The rest of the physical examination was unremarkable.

Routine blood test, urinalysis, stool routine examination, renal function, and liver function were unremarkable. The antinuclear antibodies titer was 1:100, and the rest of the autoimmune serological tests were negative. Antineutrophil cytoplasmic antibody‐associated vasculitis was negative. Tumor markers (carcinoembryonic antigen, cytokeratin 19 fragment, neuron‐specific enolase, squamous cell carcinoma antigen, and pro‐gastrin releasing peptide) were all within normal ranges. The whole blood IFN‐γ release assay was negative. The echocardiography was normal. Pulmonary function test showed a moderate obstructive ventilatory defect with normal carbon monoxide diffusing capacity.

The CT scan showed large bilateral hilar masses, the diameter of the largest mass was about 9.8 cm, and contrast‐enhanced scan showed uneven enhancement of the masses (Figure 1). Both lungs were scattered with ground glass opacities, consolidation, and nodules of varying sizes, and these lesions were mainly located in the upper lobes of both lungs. The bone destruction of the T9 vertebral body was observed.

**FIGURE 1:**
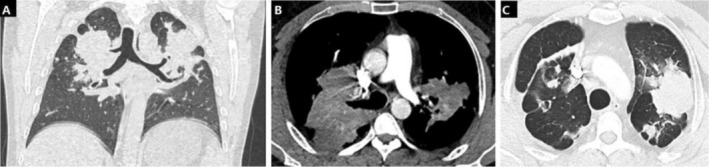
(A) Chest CT showing large bilateral hilar masses. (B) Contrast‐enhanced scan showing uneven enhancement of the masses. (C) Chest CT showing scattered ground glass opacities, consolidation, and nodules.

We performed 18F‐FDG PET/CT scanning, which indicated remarkable accumulation in the masses and a maximal standardized uptake value of 17.76 (Figure 2). The pulmonary nodules had mildly fluorine‐18 fluorodeoxyglucose (FDG) avidity (maximal standardized uptake value = 4.16), but there was no hypermetabolic lesion in the T9 vertebral body.

**FIGURE 2:**
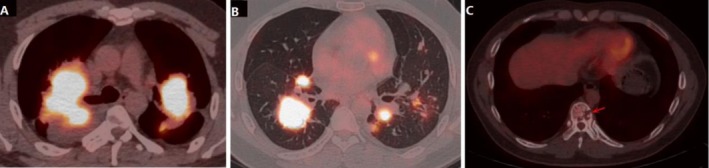
(A) PET/CT displaying large bilateral hilar masses and intensively FDG uptake with SUVmax of 17.76. (B) PET/CT displaying multiple lesions of varying size with increased FDG uptake. (C) PET/CT displaying bone destruction of the T9 vertebral body (arrow), with no significant increase in FDG uptake.

Flexible electronic bronchoscopy was performed, and round, smooth lesions were found with the complete obstruction of the bronchial lumen of the lingual bronchus of the left upper lobe and the anterior segment of the right upper lobe (Figure 3). The lumen of the posterior segment of the right upper lobe bronchus was narrow. Results of the acid‐fast bacilli smear were negative.

**FIGURE 3:**
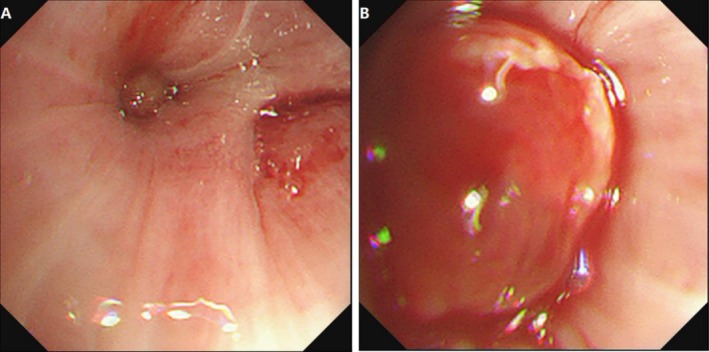
(A) Bronchoscopy revealed the obstructing endobronchial lesion of the anterior segmental bronchus of the right upper lobe and the narrow posterior bronchus. (B) Bronchoscopy revealed a round, smooth lesion, which completely obstructed the bronchial lumen of the lingual bronchus of the left upper lobe.

A biopsy specimen from the lesion of the lingual bronchus was compatible with Langerhans cell histiocytosis (LCH) (Figure 4). A histologic evaluation of the specimen demonstrated the aggregation of Langerhans cells. Immunohistochemical staining was positive for CD68, CD163, S‐100 protein, CD1a, and Langerin, and was negative for ALK, PCK, PAS, and Gomori methenamine silver.

**FIGURE 4:**
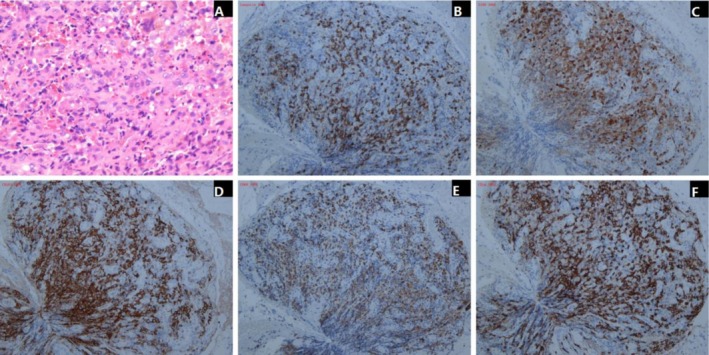
Histopathology results confirming diagnoses. (A) Representative specimen, showing the aggregation of Langerhans cells. Immunohistochemical results showing positive staining for Langerin (B), S‐100 protein (C), CD163 (D), CD68 (E), and CD1a (F).

After discharge, the patient was transferred to Peking Union Medical College Hospital. Bronchoscopy with endobronchial biopsy at the anterior segmental bronchus of the right upper lobe was performed. The pathological result was still PLCH. The patient quit smoking but refused any medication treatment, including glucocorticosteroids, chemotherapy, and targeted therapy. After quitting smoking, the patient's hemoptysis and dyspnea gradually improved. At the 1‐year, 9‐month follow‐up after discharge, he had no hemoptysis or dyspnea but still experienced intermittent coughing.

## 3 Discussion

LCH, whose formal name is histiocytosis X, is a histiocytic disorder with unknown etiology. Within the revised 2016 Histiocyte Society classification, it is now considered an inflammatory myeloid neoplasm for it has dual characteristics of inflammation and tumor, and the pathogenesis is believed to originate from myeloid dendritic cells [1, 2]. PLCH is a rare form of LCH with a strong affiliation with smoking in adults of 20–40 years of age. The clinical presentation is variable, from being minimally symptomatic to progressive respiratory failure [3].

On chest CT scan, the most common manifestations of PLCH are nodules and cysts [4]. When the presence of cysts and nodules involving middle‐upper lung zones with costophrenic sparing is observed, HRCT may be diagnostic in PLCH if clinically consistent. In the earlier stages, the typical manifestation is a centrilobular pattern with ill‐defined nodules (usually measuring from a few millimeters up to 1 cm) distributed around small airways. PLCH nodules are generally solid, but they may cavitate with time. In advanced stages, the cysts tend to predominate. Cystic changes vary in shape, size, and wall thickness. The cysts are often irregular and < 10 mm in diameter. Other radiological symptoms of smoking‐related diseases (e.g., ground glass opacities) are often visible in patients with PLCH. Pulmonary consolidation is an unusual manifestation of PLCH. In 1986, Pomeranz et al. [5] reported that a patient's chest X‐ray (not CT) showed lingular consolidation, which was confirmed by surgical lung biopsy as PLCH.

Obstructing tracheobronchial lesion is the rare manifestation of PLCH. O'Donnell et al. [6] reported the first case of endobronchial PLCH causing localized airway obstruction and left atelectasis in 1987, when PLCH was called eosinophilic granuloma of the lung. In 2005, Fridlender et al. [7] reported the first case of PLCH confined to the trachea, causing partial obstruction of the trachea. Occasionally, the endobronchial nodule is part of a more diffuse involvement [8]. It can also present with diffusely distributed lesions in the trachea and bilateral main bronchi [9]. The least common imaging manifestation of PLCH may be a mass. If a mass is observed on radiological images, clinicians need to be vigilant of concurrent bronchogenic carcinoma [10], bronchogenic carcinoma developing during a long‐term course of PLCH [11], Langerhans cell sarcoma arising from LCH [12], or coexisting with pulmonary amyloidosis [13]. The CT scan of this patient 18 months ago showed multiple masses in both lungs. At that time, bronchoscopy did not show any masses or nodules. The endobronchial lesions discovered during this bronchoscopy are believed to be formed by pulmonary parenchymal masses invading the bronchi. Biopsy performed at the left and right bronchi showed the same pathological results (PLCH). Biopsy of bilateral endobronchial lesions can clinically represent the nature of pulmonary parenchymal masses. Additionally, as of now, the patient's disease course has lasted for 3 years and 3 months, and symptoms improved after smoking cessation—both of which are consistent with the features of PLCH. The key points for differentiating between LCH and Langerhans cell sarcoma are shown in Table 1.

**TABLE 1:** Differential features between LCH and Langerhans cell sarcoma (LCS) [14, 15, 16, 17].

Feature	LCH	LCS
Histological features	Abundant eosinophils, multinucleated cells commonly seen	Marked cellular atypia/marked pleomorphism of tumor cells
Immunophenotype		
S‐100	Positive	Positive or weakly positive
CD1a	Positive	Positive or weakly positive
Ki67	10%–30%	10%–60%
Molecular features		
BRAF V600E mutation	Present (approx. 50%–60%)	Present in some cases

Abnormal PET/CT scan findings of PLCH included foci of increased uptake in nodules, consolidations, and thick‐walled cysts, especially in the early clinical course. Thin‐walled cysts appear less likely to have abnormal PET/CT scan findings. PET/CT scan imaging cannot reliably distinguish between the benign lesions of PLCH and carcinoma lesions, as both can manifest as hypermetabolic lesions [18]. PET/CT is rarely used to diagnose PLCH, but it is effective for staging and evaluating responses to therapy [19]. It can comprehensively assess the range of LCH involvement in the whole body and reveal active or inactive lesions. Regardless of whether LCH is in remission or progress, metabolic changes precede morphological changes to curative effect. PET/CT shows an advantage over conventional imaging in the survey and efficacy evaluation of multisystemic or multifocal LCH.

Our case also has some limitations. First, as a retrospective case analysis, spinal infiltration was not confirmed by MRI, MRI scans of the brain and pituitary gland were not performed, and plethysmography and the 6‐min walk test were not conducted. Additionally, the patient's symptoms resolved after smoking cessation, but the patient declined follow‐up chest CT scans, resulting in a lack of comparative imaging data.

In summary, we have reported a case of PLCH presenting as multiple obstructing bronchial lesions and pulmonary masses. PLCH needs to be considered in the differential diagnosis of multiple obstructing bronchial lesions and pulmonary masses.

## Author Contributions

Danxiong Sun designed the report. Jingyuan Fan and Chen Liu collected the patient's clinical data and wrote the manuscript. Zhen Yang analysed the imaging report. Danxiong Sun edited the manuscript. All the authors approved the final version of the article to be published.

## Funding

This work is supported by the Open Project of Yunnan Provincial Respiratory Disease Clinical Medical Center (2023YJZX‐HX10) and Open Project of Yunnan Provincial Clinical Medical Research Center for Respiratory Diseases (2026YJZX‐HX18).

## Ethics Statement

Our case report was approved by the Ethics Committee of the First People's Hospital of Yunnan Province.

## Conflicts of Interest

The authors declare no conflicts of interest.

## Data Availability

Research data are not shared.
